# Dysautonomia and small fiber neuropathy in post-COVID condition and Chronic Fatigue Syndrome

**DOI:** 10.1186/s12967-023-04678-3

**Published:** 2023-11-15

**Authors:** N. Azcue, R. Del Pino, M. Acera, T. Fernández-Valle, N. Ayo-Mentxakatorre, T. Pérez-Concha, A. Murueta-Goyena, J. V. Lafuente, A. Prada, A. López de Munain, G. Ruiz Irastorza, D. Martín-Iglesias, L. Ribacoba, I. Gabilondo, J. C. Gómez-Esteban, B. Tijero-Merino

**Affiliations:** 1 Neurodegenerative Diseases Group, Biocruces Bizkaia Health Research Institute, Plaza de Cruces 12, 48903 Barakaldo (Bizkaia), CP Spain; 2 Department of Neurology, Cruces University Hospital-OSAKIDETZA, Barakaldo, Spain; 3 Department of Neurosciences, University of the Basque Country UPV/EHU, Leioa, Spain; 4 Department of Immunology, Donostia University Hospital-OSAKIDETZA, San Sebastián, Spain; 5 Department of Neurology, Donostia University Hospital-OSAKIDETZA, San Sebastián, Spain; 6 Department of Neurosciences, Biodonostia Health Research Institute, San Sebastián, Spain; 7 Autoimmune Diseases Research Unit, Biocruces Bizkaia Health Research Institute, Barakaldo, Spain; 8 Department of Internal Medicine, Cruces University Hospital, Barakaldo, Spain; 9 The Basque Foundation for Science, IKERBASQUE, Bilbao, Spain; 10 Spanish Network for the Research in Multiple Sclerosis, San Sebastian, Spain; 11 Department of Neurosciences, University of the Basque Country UPV-EHU, San Sebastián, Spain; 12 Department of Medicine, School of Medicine, University of Deusto, Bilbao, Spain; 13 CIBERNED-CIBER, Institute Carlos III, Madrid, Spain; 14 Department of Autoimmune Diseases, Cruces University Hospital-OSAKIDETZA, Barakaldo, Spain

**Keywords:** Cognition, Dysautonomia, Myalgic Encephalomyelitis/Chronic Fatigue Syndrome, Post-COVID condition, Small fiber neuropathy

## Abstract

**Background:**

Myalgic Encephalomyelitis/Chronic Fatigue Syndrome (ME/CFS) and post-COVID condition can present similarities such as fatigue, brain fog, autonomic and neuropathic symptoms.

**Methods:**

The study included 87 patients with post-COVID condition, 50 patients with ME/CFS, and 50 healthy controls (HC). The hemodynamic autonomic function was evaluated using the deep breathing technique, Valsalva maneuver, and Tilt test. The presence of autonomic and sensory small fiber neuropathy (SFN) was assessed with the Sudoscan and with heat and cold evoked potentials, respectively. Finally, a complete neuropsychological evaluation was performed. The objective of this study was to analyze and compare the autonomic and neuropathic symptoms in post-COVID condition with ME/CFS, and HC, as well as, analyze the relationship of these symptoms with cognition and fatigue.

**Results:**

Statistically significant differences were found between groups in heart rate using the Kruskal–Wallis test (H), with ME/CFS group presenting the highest (H = 18.3; *p* ≤ *.*001). The Postural Orthostatic Tachycardia Syndrome (POTS), and pathological values in palms on the Sudoscan were found in 31% and 34% of ME/CFS, and 13.8% and 19.5% of post-COVID patients, respectively. Concerning evoked potentials, statistically significant differences were found in response latency to heat stimuli between groups (H = 23.6; *p* ≤ *.*01). Latency was highest in ME/CFS, and lowest in HC. Regarding cognition, lower parasympathetic activation was associated with worse cognitive performance.

**Conclusions:**

Both syndromes were characterized by inappropriate tachycardia at rest, with a high percentage of patients with POTS. The prolonged latencies for heat stimuli suggested damage to unmyelinated fibers. The higher proportion of patients with pathological results for upper extremities on the Sudoscan suggested a non-length-dependent SFN.

**Supplementary Information:**

The online version contains supplementary material available at 10.1186/s12967-023-04678-3.

## Introduction

Post-COVID condition (RA02 in ICD-11), also known as *long covid*, and Myalgic Encephalomyelitis or Chronic Fatigue Syndrome (ME/CFS) (8E49 in ICD-11) are characterized by unusual fatigue, cardiovascular events such as arrhythmias, palpitations and increased heart rate (HR), myalgia, headaches, non-restorative sleep, post-exertional malaise, and cognitive problems such as “brain fog” [1–5].

In post-COVID condition, these symptoms must persist for at least 12 weeks after infection [5], whereas in ME/CFS symptoms must be present for at least 6 months [3]. While in post-COVID the cause is clear, the precipitating factor for ME/CFS is not. It is usually a gastrointestinal or respiratory infection, such as the Epstein-Barr virus, Cytomegalovirus, or Borrelia burgdorferi, or a stressful live event [4].

The main symptom that both pathologies share, and which cause is still unknown, is fatigue. Other studies have previously attempted to understand the cause of fatigue in other diseases with neurological involvement. Fatigue is an independent symptom whose cause is still unclear [6–8]. The involvement of the autonomic nervous system (ANS) in the physiological activation and sensation of fatigue has been previously highlighted, and dysautonomia could be one of the causes [9]. The presence of dysautonomia in patients with post-COVID condition, and patients with ME/CFS has already been described [10, 11], being Postural Orthostatic Tachycardia (POTS) the most common autonomic syndrome [10, 12, 13]. POTS is characterized by inappropriate tachycardia in standing position, which is accompanied by dizziness, visual blurring, weakness, general malaise, and brain fog [12, 14, 15].

Small fiber neuropathy (SFN) has been suggested to be a possible mediator of POTS [16]. SFN can affect both autonomic and sensory fibers [17]. The involvement of autonomic SFN can produce changes in sweating, dry mouth, and gastrointestinal and urinary problems, as well as POTS [17]. Sensory SFN can cause paresthesia, burning sensation, pain, or allodynia [17, 18]. Most patients with SFN experience sensory disturbances that start in the feet and progress upwards, affecting the fibers progressively starting with more distal areas. This type of SFN is called length-dependent SFN. Neuropathy can also be non-length-dependent; in which cases, symptoms are “patchy” and fluctuate over time [19]. Many factors can contribute to the development of SFN such as infections, metabolic disorders, toxic agents, or inflammatory processes [17, 18]. Both patients with post-COVID-19 condition and ME/CFS could develop these types of neuropathies [20–22].

Therefore, this study aims to analyze the similarities and differences between these two types of patients, analyzing the presence of dysautonomia and SFN in ME/CFS and post-COVID-19 condition, and their possible relationship with other symptoms, such as cognition or fatigue, comparing these two types of patients with healthy controls (HC).

## Materials and methods

### Participants and demographic data

We recruited 87 participants with post-COVID condition, and 50 patients with ME/CFS at the Neurology Department of Cruces University Hospital, and 50 HC with a mean of age of 44.1 ± 8.5, 45.6 ± 9.4, 42.3 ± 9.9, respectively. Sex, years of education and disease duration, dysautonomic, neuropathic, and neuropsychiatric symptoms were recorded for all participants (Table 1).

**Table 1:** Sociodemographic data and clinical status

	HC (n = 50) M (SD)	ME/CFS (n = 50) M (SD)	Post-COVID (n = 87) M(SD)	Statistics	Bonferroni (*p*)		
					ME/CFS vs post-COVID	ME/CFS vs HC	Post-COVID vs HC
Age, years	42.3 (9.9)	44.1 (8.5)	45.6 (9.4)	H = 3.6			
Education, years	16.7 (2.5)	14.59 (4.1)	16.3 (3.3)	H = 10.7**	0.042	0.004	
Female, *n* (%)	40 (80.0)	45 (90.0)	62 (71.3)	*χ*2 = 6.7*			
Disease duration, months	–	52.4 (50.4)	13.7 (6.9)	U = 3696.0***			
COMPASS 31	3.8 (4.6)	25.4 (10.5)	19.6 (8.9)	H = 96.2***	0.039	< 0.001	< 0.001
SFNSL	2.1 (3.4)	34.7 (15.7)	26.6 (14.4)	H = 96.8***		< 0.001	< 0.001
Karnofsky scale	99.0 (3.6)	68.9 (8.8)	73.1 (8.4)	H = 118.4***		< 0.001	< 0.001
SF-36	85.7 (9.7)	29.1 (15.0)	40.4 (17.0)	H = 108.5***	0.016	< 0.001	< 0.001
MFIS	10.5 (11.8)	68.1 (12.8)	63.4 (15.2)	H = 106.8***		< 0.001	< 0.001
PSQI	5.1 (3.0)	12.6 (5.0)	10.7 (4.5)	H = 59.2***		< 0.001	< 0.001
STAI-State	11.9 (10.2)	30.2 (16.0)	27.3 (13.9)	H = 44.9***		< 0.001	< 0.001
STAI-Trait	13.0 (9.4)	24.6 (16.0)	16.4 (13.6)	H = 14.7***	0.007	0.001	
GDS	1.1 (11.5)	9.3 (3.4)	7.0 (3.8)	H = 95.1***	0.025	< 0.001	< 0.001
C-SSRS	0.0 (0.0)	1.2 (1.9)	0.2 (0.5)	H = 34.1***	< 0.001	< 0.001	

^*^p ≤ 0.05; **p ≤ 0.01; ***p ≤ 0.001. COMPASS: The Composite Autonomic Symptom Score; C-SSRS: Columbia Suicide Severity Rating Scale; GDS: Geriatric Depression Scale; H: Kruskal–Wallis test; HC: healthy controls; ME/CFS: Myalgic Encephalomyelitis/Chronic Fatigue Syndrome, MFIS: Modified Fatigue Impact Scale; PSQI: Pittsburgh Sleep Quality Index; SF-36: The 36-Item Short Form Health Survey; SFNSL: Small Fiber Neuropathy Screening List; STAI: State-Trait Anxiety Inventory

The inclusion criteria encompass participants between 18–85 years old, with a sufficient understanding and communication skills. Participants with pregnancy and/or lactation, severe trauma, alcoholism, drug addiction, severe heart disease, radiological diagnosis of brain structural pathology, concomitant diseases that could influence the results, as well as patients who have received some immunomodulatory treatments were excluded. Patients diagnosed with post-COVID condition met the criteria proposed by the NICE guidelines, in which signs and symptoms that develop during or after the infection consistent with COVID-19 continued for more than 12 weeks and were not explained by an alternative diagnosis [23]. For the diagnosis of acute COVID-19, the valid diagnostic methods were a positive nasal PCR, the detection of IgG and/or IgM antibodies against SARS-CoV-2 or a medical report supporting the diagnosis [5]. Exclusion criteria in this group included respiratory disease lasting 12 weeks after the infection, having been admitted to an intensive care unit and/or having had severe bilateral pneumonia or other severe disease manifestations requiring hospitalization. Patients diagnosed of ME/CFS should be previously diagnosed or meet the Fukuda et al. [3] criteria at the evaluation time.

The study protocol was approved by the Basque Drug Research Ethics Committee [*Comité de Ética de la Investigación con medicamentos de Euskadi* (CEIm-E) (PI2020210)]. All participants gave written informed consent prior to their participation in the study, in accordance with the tenets of the Declaration of Helsinki.

### Autonomic function test assessment

All patients and HC included in the studied were evaluated about autonomic symptoms using the Composite Autonomic Symptom Score (COMPASS-31) that assesses the patient-reported manifestations across six dimensions of autonomic function: orthostatic intolerance, sudomotor, vasomotor, gastrointestinal, bladder, and pupillomotor domains.

Non-invasive quantitative measures for hemodynamic autonomic function were determined with a Task Force Monitor (CNSystems, Graz, Austria) following standard procedures for quantitative autonomic testing [24]. HR and blood pressure (BP) variability were continuously monitored during the hemodynamic autonomic evaluation. Firstly, values were obtained at rest in a supine position for 10 min. Four-lead electrocardiography was used to measure the HR and inter-beat interval. Secondly, cardiovagal function was evaluated with the deep breathing test and the sympathetic system by the Valsalva maneuver. The expiratory-to-inspiratory ratio (E/I) and deep breathing index were calculated using results from the deep breathing test, which consisted of six successive deep breath cycles in supine position. This was followed by the Valsalva maneuver performed at an expiratory pressure of 40 mmHg for 15s. Blood pressure recovery time (PRT) was calculated which is the recovery time of systolic blood pressure (sBP) from the bottom of phase III to baseline, and the Valsalva ratio calculated with the maximum HR divided by the lowest HR generated by the Valsalva maneuver. Thirdly, values were obtained in a 11-min Tilt Test at 60º. POTS was defined as an excessive orthostatic tachycardia in the absence of substantial orthostatic hypotension and a HR increase of ≥ 30 bpm or above 120 bpm within first 5 min after assuming standing position, accompanied by symptoms of orthostatic intolerance [14]. All autonomic function tests were carried out by experienced neurologists.

### Small fiber function assessment

SFN symptoms were assessed using Small Fiber Neuropathy Screening List (SFNSL) consisting of 21 questions.

The functioning of nerve signals from ANS that controls sweat production was quantified non-invasively with the Sudoscan test. This device quantifies the electrochemical skin conductance (ESC) in palms and soles. ESC results are expressed in microSiemens (μS) [25].

The response capacity of the small fibers responsible for the sensory information of pain and temperature (type C and Aδ fibers) was evaluated with the TSA-2 device (Medoc Advanced Medical Systems, Ramat Yishai, Israel) which includes the contact heat evoked potentials (CHEPs), cold evoked potentials (CEPs) and quantitative sensory testing (QST). For CHEPs (C type fibers), 15 stimuli of 55ºC every 30–45 s were performed, and 15 stimuli of 9ºC every 30–45 s for CEPs (Aδ fibers). Values for latency (ms) and amplitude (μV) were obtained.

### Cognitive and neuropsychiatric assessment

A comprehensive battery of neuropsychological and neuropsychiatric tests was used to evaluate specific cognitive domains and neuropsychiatric status: general cognition, processing speed, verbal fluency, attention, verbal memory, visual memory, visuoconstructive ability, visuospatial ability, abstraction, executive functions, anxious-depressive symptoms, general health perception, fatigue level, and sleep quality. Neuropsychological and neuropsychiatric evaluations were performed by experienced neuropsychologists (Additional file 1).

### Statistical analysis

Statistical analyses were carried out with IBM SPSS Statistics for Windows, 26.0 version (IBM SPSS, Armonk, NY, USA). The assumptions of normality and homogeneity of variances of the variables were analyzed. Group differences for continuous and categorical variables were analyzed with Kruskal–Wallis and Chi square test, respectively. Kruskal–Wallis statistics were adjusted with Bonferroni corrections for multiple comparisons. The Mann–Whitney U test was used to analyze differences in disease duration between post-COVID and ME/CFS groups. Cognitive composites were created by averaging z-scores of cognitive tests in each domain. Spearman bivariate correlations were calculated to analyze the relationship of autonomic variables with small fiber assessment parameters and with cognitive performance. Finally, ROC curve analysis was performed to determine the variables that best discriminated patients from HC. The cut-off point of the Area Under the Curve (AUC) was established at AUC ≥ 0.75, highest sensitivity and specificity point was calculated with the Youden Index (J). Statistical significance was set at* p* < 0.05 (two-tailed).

## Results

### Demographic and clinical data

Demographic and clinical data are shown in Table 1. No significant differences were found between groups for age. Differences in gender distribution were found between ME/CFS and post-COVID groups (χ2 = 6.51, *p* = 0.011), with more women in the ME/CFS group (90%), followed by HC (80%), and finally by post-COVID patients (71.3%). Statistically significant differences were also found between groups in disease duration, with the ME/CFS having a longer disease duration (U = 3696.0, *p* ≤ 0.001).

### Autonomic nervous system

The autonomic symptoms measured through the COMPASS-31 showed significant differences between post-COVID and ME/CFS patients (*p* = 0.039), and between patients and HC (*p* < 0.001). The ME/CFS group presented more autonomic symptoms than post-COVID patients (Table 1).

**Fig. 1:**
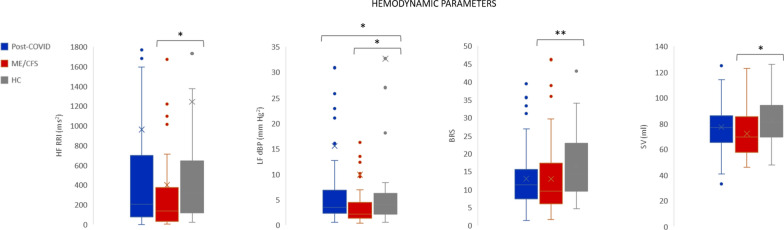
Autonomic Nervous System and hemodynamic parameters. The mean is indicated with an X, the median is represented with a line inside the box. *p ≤ 0.05; **p ≤ 0.01; BRS: baroreflex sensitivity; dBP: diastolic blood pressure; HC: healthy controls; HF: high frequency; LF: low frequency; ME/CFS: Myalgic Encephalomyelitis/Chronic Fatigue Syndrome; RRI: R-R interval; SV: stroke volume

Spectral analysis of HR and BP variability were used to analyze the sympathetic and parasympathetic activation and the autonomic balance (Fig. 1). Statistically significant differences were found in high frequency R-R interval (HF-RRI) between ME/CFS and HC (*p* = 0.021), in low frequency of diastolic blood pressure in normalized units (LFnu-dBP) between ME/CFS and HC (*p* = 0.032), and ME/CFS and post-COVID (*p* = 0.013) (Fig. 1). The sympathetic-parasympathetic balance (LF/HF), obtained during supine position monitoring, did not show statistically significant differences between groups. Significant differences were observed between HC and ME/CFS in stroke volume (SV) (*p* = 0.005), and baroreflex sensitivity (BRS) (*p* = 0.038), ME/CFS having lower values in both parameters.

**Fig. 2:**
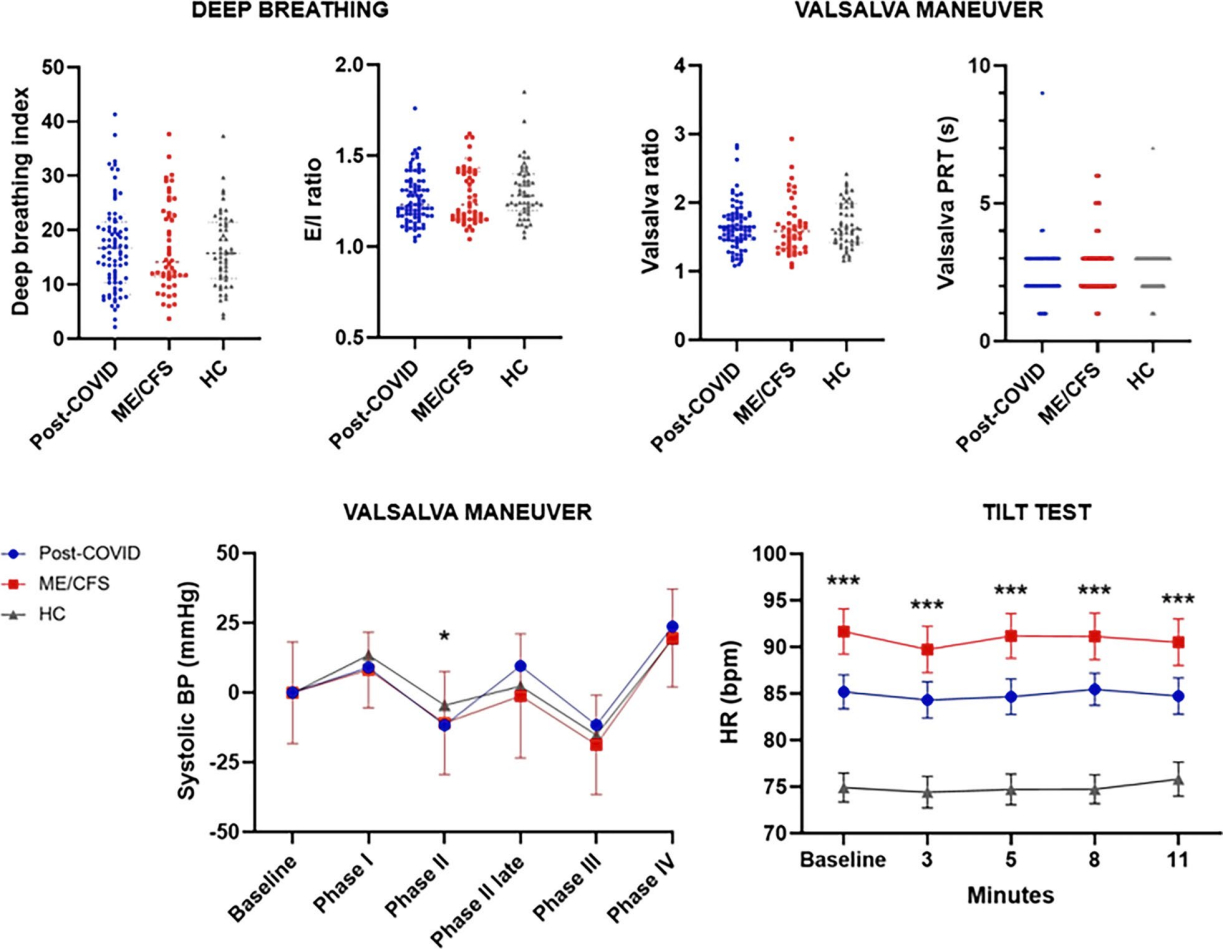
Deep breathing, Valsalva maneuver and Tilt test parameters. For Valsalva maneuver systolic blood pressure data, the differences between the baseline and each phase were taken. *p ≤ 0.05; ***p ≤ 0.001. BP: blood pressure; HC: Healthy Controls; ME/CFS: Myalgic Encephalomyelitis/Chronic Fatigue Syndrome; PRT: pressure recovery time

No significant differences between the deep breathing indexes were found between groups. A pathological E/I ratio [26] was found in 5.7% of post-COVID patients, in 4.1% of ME/CFS, and in 2% of HC. No significant differences were found in Valsalva ratio, neither in the Valsalva PRT between patients and HC. Despite not finding statistically significant differences in Valsalva PRT, the ME/CFS group showed the largest proportion of subjects with pathological PRT (14.7%), followed by post-COVID patients (5.1%) and HC (2%) (Fig. 2).

Regarding the Tilt Test, statistically significant differences were found between patients and HC (*p* ≤ 0.001), for supine and standing HR (Fig. 2). ME/CFS patients presented a 90.10 ± 15.38 basal HR, followed by post-COVID patients with 84.42 ± 15.19 and HC with 74.92 ± 11.12 HR. Likewise, 13.8% of the post-COVID patients and 31% of the ME/CFS meet the diagnostic criteria of POTS. Only one of the post-COVID patients presented a pure vasodepressor syncope, and none of the HC presented a pathological Tilt Test.

### Small fiber neuropathy

The small fiber neuropathy symptoms assessed with SFNSL showed significant differences between patients and HC (*p* < 0.001) (Table 1). No significant differences were found between groups in ESC on the Sudoscan. The 19.5% of post-COVID patients had pathological ESC in palms, and 11.5% in soles. Among the patients with ME/CFS, 34% had pathological values in palms and 12% in soles, while 18% of HC had a pathological result in palms and 8% in soles (Table 2).

**Table 2:** Quantitative autonomic testing

	HC (*n* = 50) M (SD)	ME/CFS (*n* = 50) M (SD)	Post-COVID (*n* = 87) M (SD)	Statistics	Bonferroni (*p*)		
					ME/CFS vs post-COVID	ME/CFS vs HC	Post-COVID vs HC
HF-RRI (ms2)	12,443.3 (3668.1)	399.5 (669.3)	962.5 (2986.3)	H = 7.5*		0.019	
HFnu-RRI (%)	40.9 (16.6)	37.9 (9.7)	37.5 (16.4)	H = 1.5			
LF-dBP (mmHg2)	32.6 (161.1)	9.8 (16.6)	15.5 (49.4)	H = 9.5**	0.032	0.013	
LFnu-dBP (%)	43.5 (13.1)	43.9 (14.4)	45.7 (14.3)	H = 2.3			
LF/HF	1.5 (1.0)	2.4 (3.2)	2.3 (2.6)	H = 3.5			
SV	82.4 (16.6)	72.2 (18.1)	77.3 (17.3)	H = 10.1**		0.005	
CO	5.3 (1.3)	5.4 (1.5)	5.7 (1.4)	H = 2.9			
TPR	1400.9 (427.4)	1381.5 (344.0)	1405.4 (465.4)	H = 0.2			
BRS mean	16.3 (8.6)	12.9 (9.7)	13.0 (7.9)	H = 7.4*		0.038	
Deep breathing index	16.3 (6.9)	16.8 (8.2)	16.8 (8.0)	H = 0.0			
E/I ratio	1.3 (0.1)	1.2 (0.1)	1.2 (0.1)	H = 1.9			
Valsalva ratio	1.7 (0.3)	1.6 (0.4)	1.6 (0.3)	H = 0.7			
Valsalva PRT (s)	2.6 (0.9)	2.8 (1.1)	2.5 (1.0)	H = 1.9			
ΔsBP phase II	– 4.5 (19.7)	– 11.0 (18.5)	– 11.7 (22.4)	H = 7.5*			0.035
ΔdBP phase II	– 33.1 (19.8)	– 33.7 (15.2)	– 33.7 (16.6)				
ΔsBP phase II late	2.2 (20.3)	– 1.2 (22.2)	– 0.6 (19.8)				
ΔdBP phase II late	12.8 (15.3)	8.7 (15.1)	12.4 (18.1)				
ΔsBP phase IV	19.2 (17.4)	19.5 (17.5)	23.6 (18.8)				
ΔdBP phase IV	10.7 (13.0)	10.5 (9.9)	15.7 (15.4)				
*Hemodynamic responses to Tilt*							
Supine							
sBP	112.7 (21.0)	112.6 (18.)	112.6 (13.5)	H = 0.9			
dBP	72.8 (13.3)	75.9 (11.9)	74.2 (12.8)	H = 2.7			
HR	65.7 (9.8)	76.2 (13.5)	73.4 (12.4)	H = 18.3***		< 0.001	0.001
3 min							
sBP	129.9 (28.6)	126.6 (18.4)	126.0 (15.3)	H = 0.0			
dBP	89.7 (16.5)	89.2 (15.0)	88.5 (14.9)	H = 1.9			
HR	74.4 (12.1)	89.7 (17.7)	84.3 (18.1)	H = 20.2***		< 0.001	0.004
5 min							
sBP	125.6 (23.3)	125.6 (20.2)	122.6 (18.9)	H = 0.5			
dBP	85.5 (17.8)	89.2 (15.0)	86.0 (13.6)	H = 2.2			
HR	74.7 (11.7)	91.1 (16.9)	84.6 (17.6)	H = 23.9***		< 0.001	0.004
8 min							
sBP	124.8 (19.6)	122.0 (24.0)	121.9 (15.2)	H = 0.4			
dBP	84.4 (15.2)	88.1 (14.7)	84.3 (16.7)	H = 1.7			
HR	74.7 (10.9)	91.1 (17.4)	85.4 (15.9)	H = 25.2***		< 0.001	< 0.001
11 min							
sBP	124.7 (20.7)	121.7 (18.7)	120.7 (19.9)	H = 0.2			
dBP	83.3 (16.6)	86.5 (15.3)	84.8 (13.7)	H = 1.8			
HR	75.8 (12.9)	90.5 (16.2)	84.7 (17.9)	H = 19.6***		< 0.001	0.002
*Mean values (Tilt)*							
sBP	126.3 (20.8)	128.9 (38.3)	129.4 (43.4)	H = 0.1			
dBP	85.7 (14.7)	88.5 (13.7)	88.6 (27.4)	H = 0.9			
HR	74.9 (11.0)	91.6 (17.2)	85.1 (17.0)	H = 26.4***		< 0.001	0.001

For Valsalva maneuver blood pressure data, the differences between the baseline and each phase were taken. *p ≤ 0.05; **p ≤ 0.01; ***p ≤ 0.001. BRS: baroreflex sensitivity; CO: cardiac output; dBP: diastolic blood pressure; H: Kruskal–Wallis test; HC: healthy controls, HF: high frequency; HR: heart rate; LF: low frequency; ME/CFS: Myalgic Encephalomyelitis/Chronic Fatigue Syndrome; nu: normalized units; PRT: pressure recovery time; RRI: R-R interval; sBP: systolic blood pressure; SV: stroke volume; TPR: total peripheral resistance

**Fig. 3:**
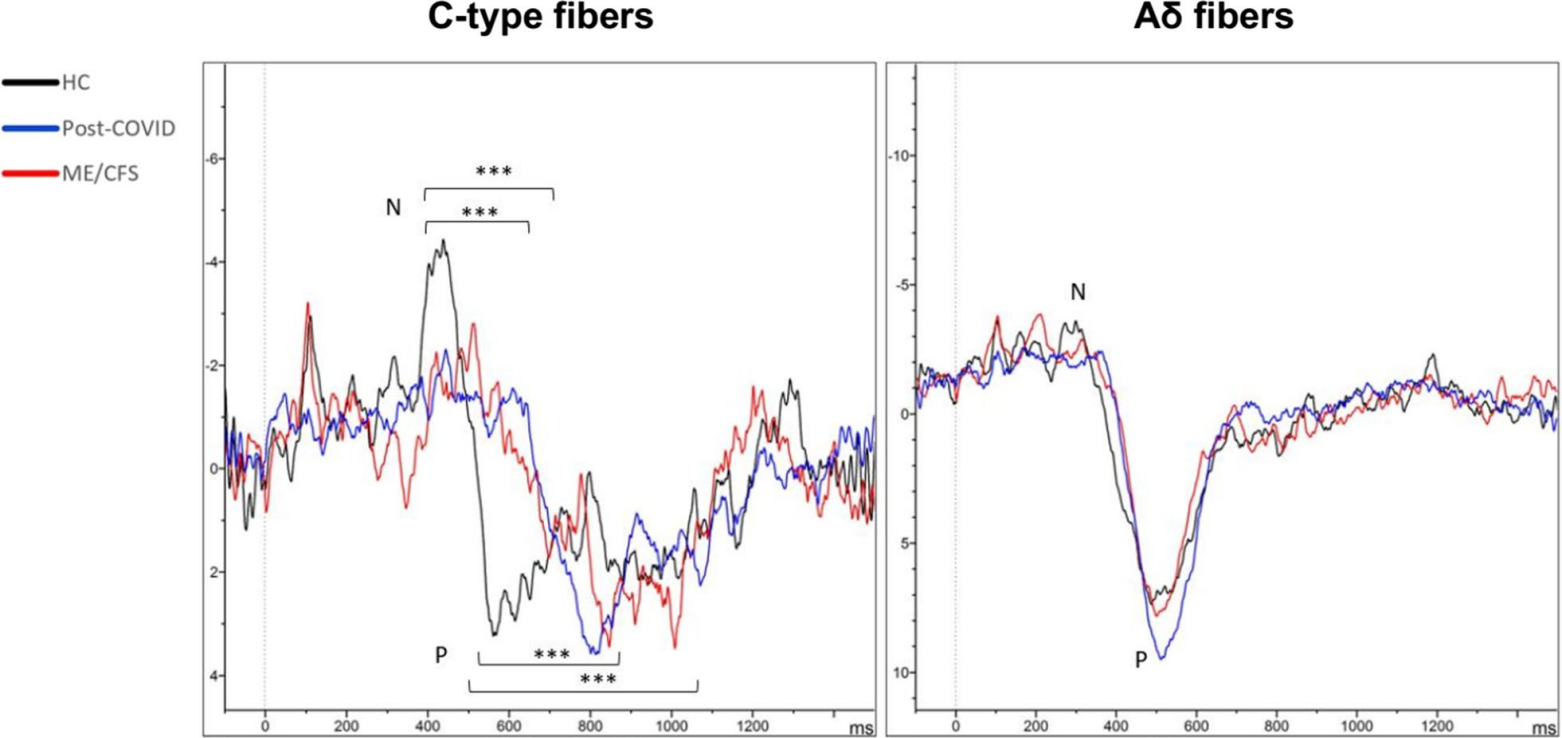
Heat and cold evoked potentials*.* The results shown are the mean of each group for the different tests. N refers to first definitive negative peak, and P to the first definitive positive peak after the trigger. ***p ≤ 0.001. HC: Healthy Controls; ME/CFS: Myalgic Encephalomyelitis/Chronic Fatigue Syndrome

As to QST, statistically significant differences were found in the ability to detect heat between HC and post-COVID patients (*p* = 0.001), with HC having lower thresholds for temperature change detection. Regarding CHEPs, significant differences in the latency of the N wave between HC and patients were found (*p* < 0.001) (Table 3). Response latency was larger in ME/CFS patients (686 ± 16), followed by post-COVID patients (676 ± 15), and finally by HC (552 ± 13) (Fig. 3). Differences in P wave latency between patients and HC were also found (*p* = 0.001) (Table 3). No significant differences were found in the responses to cold stimuli, neither in latency nor in the amplitude (Fig. 3).

**Table 3:** Small fiber assessment

Small fiber assessment	HC (*n* = 50) M (SD)	ME/CFS (*n* = 50) M (SD)	Post-COVID (*n* = 87) M (SD)	Statistics (H)	Bonferroni (*p*)	
					ME/CFS vs HC	Post-COVID vs HC
*Sympathetic small fibers*						
***Sudoscan ESC***						
Feet (μS)	72.9 (12.5)	70.8 (14.3)	74.3 (13.2)	3.4		
Hands (μS)	72.4 (10.1)	66.8 (16.5)	71. (16.3)	2.8		
*Sensory small fibers*						
***QST***						
Heat detection	35.6 (2.3)	36.4 (2.5)	36.9 (2.7)	13.2**		0.001
Cold detection	26.9 (3.3)	26.9 (4.5)	26.5 (3.7)	1.5		
Pain with heat	43.8 (4.8)	42.4 (4.6)	43.1 (4.7)	2.7		
Pain with cold	12.5 (9.9)	15.5 (9.3)	14.7 (10.1)	3.2		
Heat detection 2	33.5 (1.5)	34.3 (1.2)	34.9 (2.6)	28.9***	0.001	< 0.001
*Contact evoked potentials*						
***Heat***						
N latency	0.5 (0.1)	0.7 (0.1)	0.7 (0.1)	23.6**	< 0.001	< 0.001
N amplitude	– 11.2 (7.5)	– 9.3 (5.7)	– 0.7.9 (4.9)	7.3*		0.026
P latency	0.7 (0.2)	0.8 (0.2)	0.8 (0.2)	17.9**	0.001	0.001
P amplitude	12.8 (6.1)	13.3 (6.1)	12.6 (6.1)	0.6		
***Cold***						
N latency	0.3 (0.01)	0.3 (0.1)	0.3 (0.1)	0.1		
N amplitude	– 8.1 (5.4)	– 8.2 (6.7)	– 8.3 (4.3)	1.3		
P latency	0.5 (0.1)	0.5 (0.1)	0.5 (0.1)	0.1		
P amplitude	13.7 (5.9)	14.7 (7.9)	15.3 (6.7)	2.9		

In the QST, the values indicate temperature in Celsius degrees. Latencies are shown in seconds, and amplitudes are shown in microvolts. *p ≤ 0.05; **p ≤ 0.01; ***p ≤ 0.001. ESC: electrochemical skin conductance; H: Kruskal–Wallis test; HC: healthy controls; ME/CFS: Myalgic Encephalomyelitis/Chronic Fatigue Syndrome; QST: quantitative sensory testing

### Small fiber involvement in autonomic function

The correlations between autonomic and small fiber function parameters for both ME/CFS and post-COVID patients were analyzed (Additional file 1). In the post-COVID group, the better the parasympathetic functioning, the greater the ESC on the Sudoscan (Rho = 0.25, *p* = 0.018), and better heat detection (Rho = 0.26, *p* = 0.015) were found.

Similar results were found in ME/CFS patients, disease duration correlated with CO (Rho = -0.28, *p* = 0.046), E/I ratio (Rho = -0.34, *p* = 0.018), and palms ESC (Rho = -0.46, *p* = 0.001). A better parasympathetic response (E/I ratio) correlated with better ESC in palms (Rho = 0.41, *p* = 0.003), and better heat (Rho = 0.32, *p* = 0.024), and cold detection (Rho = 0.41, *p* = 0.004) in the QST. Greater sympathetic activation (sBP in Valsalva phase IV), in turn, was related to better ESC in palms (Rho = 0.39, *p* = 0.006).

### Autonomic and hemodynamic involvement in cognition and clinical status

Correlations between hemodynamic and ANS parameters with cognitive performance and neuropsychiatric variables were analyzed (Additional file 1). Lower HR during the Tilt test correlated to better cognitive performance in post-COVID patients, specifically in attention capacity (Rho = -0.32, *p* = 0.003), and processing speed (Rho = -0.36, *p* = 0.001). Fatigue levels worsened the lower palms (Rho = -0.22, *p* = 0.042) and soles ESC (Rho = -0.23, *p* = 0.037), and the lower the SV (Rho = -0.25, *p* = 0.023).

In ME/CFS patients, cognitive performance, mainly processing speed, improved the better the parasympathetic response (Rho = 0.39, *p* = 0.007). Cognitive performance, mainly the verbal memory (Rho = 0.37, *p* = 0.009), improved the higher the HR during the Tilt Test. The HR was not related to fatigue levels in either of the two groups of patients.

### Clinical features of ME/CFS and post-COVID patients

**Fig. 4:**
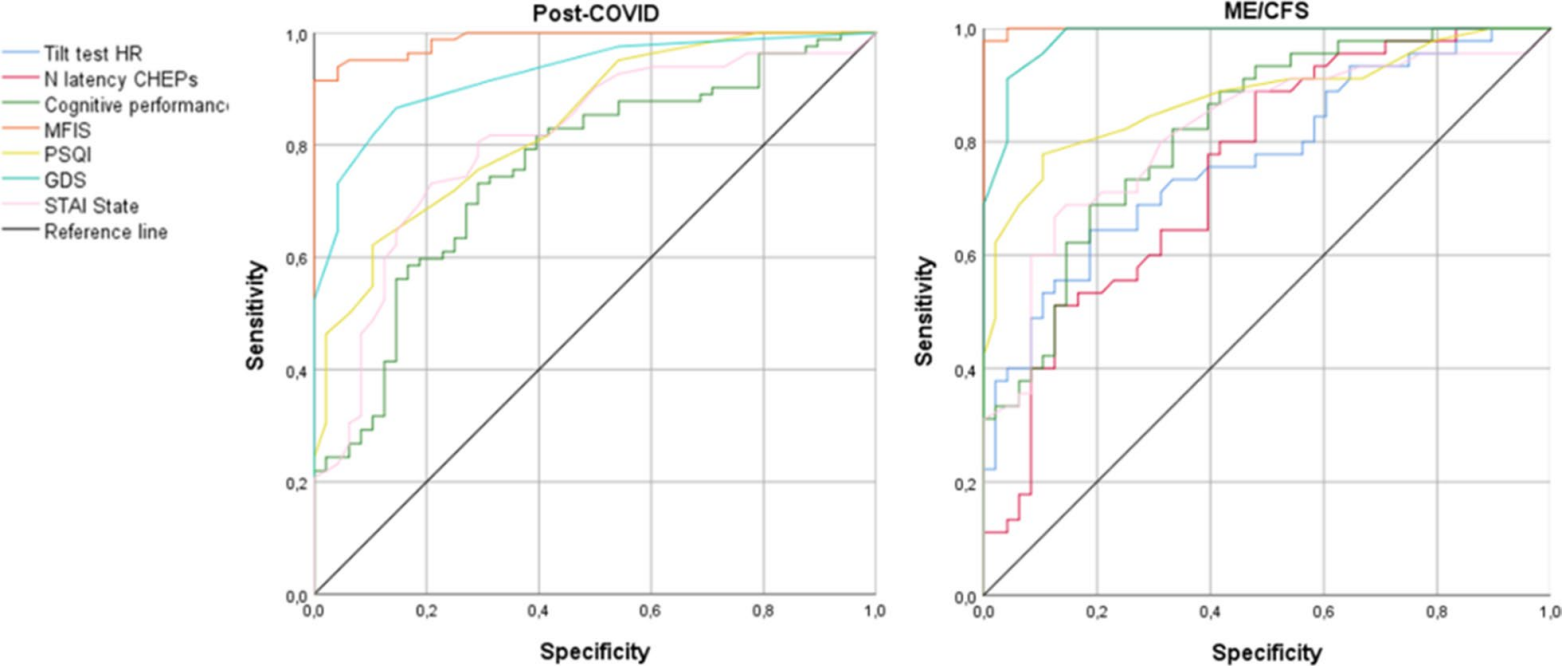
Clinical characteristics of ME/CFS and post-COVID patients compared to HC. Cognitive performance refers to the mean of all the neuropsychological tests’ performance. The Tilt test HR refers to the mean values of 3, 5, 8, and 11 min during the Tilt test. HR: heart rate; ME/CFS: Myalgic Encephalomyelitis/Chronic Fatigue Syndrome

ROC curves were performed to know the variables that best discriminated between the two groups of patients and HC (Fig. 4). The ROC curves identified cognitive performance (AUC = 0.75, *p* < 0.00), fatigue level (AUC = 0.98, *p* < 0.001), sleep quality (AUC = 0.83, *p* < 0.001), depressive symptoms (AUC = 0.92, *p* < 0.001), and anxiety symptoms (AUC = 0.80, *p* < 0.001) as variables that discriminated between HC and post-COVID patients. The tests that presented higher specificity and sensitivity in these patients were fatigue level, with a sensitivity of 91.7% and a specificity of 100% (J = 0.92), and depressive symptoms, with a sensitivity of 86.9% and a specificity of 85.7% (J = 0.73).

In ME/CFS patients, Tilt test HR (AUC = 0.77, *p* < 0.001), N latency in CHEPs (AUC = 0.75, *p* < 0.001), cognitive performance (AUC = 0.82, *p* < 0.001), fatigue level (AUC = 0.99, *p* < 0.001), sleep quality (AUC = 0.87, *p* < 0.001), depressive symptomatology (AUC = 0.98, *p* < 0.001), and anxiety symptoms (AUC = 0.81, *p* < 0.001) were the variables that best discriminated between patients and HC. The tests that presented higher specificity and sensitivity were fatigue level, with a sensitivity of 97.9% and a specificity of 100% (J = 0.98), and depressive symptoms, with a sensitivity of 91.5% and a specificity of 95.8% (J = 0.87).

## Discussion

In this study, autonomic and neuropathic symptoms and signs of post-COVID condition and ME/CFS were compared to HC, with the aim to known if both syndromes presented the same affectation. The relationship between autonomic and hemodynamic function and neuropathic symptoms was analyzed, also exploring their possible relationship with cognitive performance and fatigue. To our knowledge, this is the first study to analyze the relationship of these signs and symptoms in ME/CFS and post-COVID condition patients.

These results highlight the high prevalence of autonomic impairment in these patients and its possible implication in cognitive performance. In turn, the results suggest sensory neuropathy, so it would be advisable to perform evaluations to rule out neuropathy and dysautonomia in these patients.

### Dysautonomia and small fiber neuropathy

Post-COVID condition and ME/CFS patients often report autonomic symptoms such as tachycardia, frequent urination, dry eyes, dry mouth, digestive disturbances, blurred vision, and sensitivity to light [27, 28]. The COMPASS-31 results showed significant differences between patients and HC, with ME/CFS patients reporting more autonomic symptoms than the post-COVID group.

The evaluation of the ANS (mainly cardiovagal function), and hemodynamic parameters showed significant differences between groups in HF-RRI, LF-dBP, SV and BRS, with ME/CFS patients having the lowest values. These results indicate less variability in the response of the ANS in ME/CFS. Statistically significant differences were found between groups in HR, both in supine and during the Tilt Test between patients and HC. The causes of POTS can be diverse, including autonomic neuropathy, a hyperadrenergic state, autoimmune diseases, and cardiovascular deconditioning [29]. More specifically, POTS after COVID-19 or ME/CFS may be due to a systemic inflammatory state, a state of increased sympathetic tone driven due to autoantibodies, peripheral neuropathy producing compensatory tachycardia, or neuropathy causing a dysfunction of the parasympathetic nervous system [29]. The prevalence of POTS is around 0.2% to 1% in the general population [30, 31], finding a much higher percentage in patients with ME/CFS (31%) or post-COVID condition (13.8%) in our sample. This autonomic syndrome could be one of the causes of Brain Fog in post-COVID patients, having into account the correlations found in our study.

The functioning of small autonomic and sensory fibers was evaluated. There were not statistically significant differences between groups in ESC, although a higher percentage of patients with pathological ESC in palms than in soles was found. These results suggest that the neuropathy these patients may suffer does not follow a length-dependent pattern. Non-length-dependent small fiber neuropathy (NLD-SFN) is more common in young women. In these cases, topographic patterns are patchy and asymmetrical, and the symptoms can fluctuate [19]. Despite not finding significant differences between groups, a lower ESC in ME/CFS patients with a longer disease duration was found, suggesting a progressive deterioration of the cholinergic sweat small fibers.

Regarding sensory fibers, significant differences were found between groups for heat detection in QST and in CHEPs. ME/CFS and post-COVID patients had higher response latencies to heat stimuli than HC, not being differences between these two pathologies. These results could indicate denervation or damage of C-type unmyelinated fibers. These results are similar to those found in other studies, showing that sensory small fiber neuropathy is common in patients with post-COVID syndrome [21] and ME/CFS [22].

Concerning the relationship between the functionality of sensory and autonomic small fibers with autonomic parameters, patients without neuropathy showed better cardiovagal functioning and lower HR. Therefore, despite not finding significant differences between patients and HC in parasympathetic function, the existence of parasympathetic or cholinergic dysfunction both in ME/CFS and in post-COVID patients could be hypothesized. In fact, some studies correlate cholinergic dysfunction as a possible cause of fatigue and dysautonomia in patients with the post-COVID syndrome [32].

### Future research

The results presented in this study could be used as data to support the ME/CFS and post-COVID condition diagnosis. To do this, for future studies it would be advisable to expand the HC sample and establish the pathological thresholds for autonomic and neuropathic signs. Future studies could broad the evaluation of the ANS to determine dysautonomia signs by other techniques, such as pupillometry [33]. Likewise, more tests could be carried out to determine the existence of neuropathy, corneal confocal microscopy could be an adequate non-invasive technique for this [19, 34]. Future studies could also analyze the presence of autoantibodies to determine their possible involvement in the development of post-COVID condition and ME/CFS [35]. In this way, it could be known if the symptomatology is due to autoimmunity and guide possible treatments for this cause [35, 36].

### Limitations

The study presents various limitations. First, the sample size was not the same for the different groups. Therefore, it is possible that certain significant correlations have not been observed in patients with ME/CFS. Secondly, it must also be taken into account that the proportion of men and women is different for each group. Therefore, it could be a sexual bias, since we do not know if the symptomatologic expression of these diseases changes according to sex. It must also be taken into account that these pathologies are more prevalent in women. Finally, the high percentage of HC with pathological results in ANS must be considered when evaluating sympathetic or parasympathetic functions. The cause of these possible alterations is unknown. These results may be hiding the differences between the patients and HC in our sample.

## Conclusion

Post-COVID condition and ME/CFS patients presented a higher HR both in supine and standing position compared to HC. The number of patients with POTS in both syndromes was much higher than the expected in normal population. Autonomic tests results indicated less variability of HR in the response of the cardiovagal function in ME/CFS compared to HC, this was not found in post-COVID patients. Both syndromes had larger latencies than HC for hot stimuli in CHEPs, suggesting sensory small fiber neuropathy in upper extremities.

### Supplementary Information

**Additional file 1.** Detailed neuropsychologic assessment, and Autonomic Nervous System correlation table.

## Data Availability

Not applicable.

## Abbreviations

AUC: Area under the curve
BRS: Baroreflex sensitivity
CEPs: Cold evoked potentials
CHEPs: Contact heat evoked potentials
CO: Cardiac output
COMPASS: Composite Autonomic Symptom Score
dBP: Diastolic blood pressure
ESC: Electrochemical skin conductance
HC: Healthy controls
HF: High frequency
HR: Heart rate
LF: Low frequency
ME/CFS: Myalgic Encephalomyelitis/Chronic Fatigue Syndrome
NLD-SFN: Non-length-dependent small fiber neuropathy
nu: Normalized units
POTS: Postural Orthostatic Tachycardia Syndrome
PRT: Pressure recovery time
RRI: R-R interval
sBP: Systolic blood pressure
SFN: Small fiber neuropathy
SFNSL: Small Fiber Neuropathy Screening List
SV: Stroke volume
TPR: Total peripheral resistance
QST: Quantitative sensory testing
