# Supplementary material for: Dysautonomia and small fiber neuropathy in post-COVID condition and Chronic Fatigue Syndrome
Source: J Transl Med. 2023 Nov 15;21:814. doi: 10.1186/s12967-023-04678-3 (PMC10648633; doi:10.1186/s12967-023-04678-3)
Supplement: Supplementary file 1 — Additional file 1. Detailed neuropsychologic assessment, and Autonomic Nervous System correlation table. [file 12967_2023_4678_MOESM1_ESM.docx]

**Dysautonomia and small fiber neuropathy in post-COVID condition and Chronic Fatigue Syndrome**

Azcue, N^1^; Del Pino, R.^1^*; Acera, M.^1^; Fernández-Valle, T.^1,2,3^; Ayo-Mentxakatorre, N.^1^, Pérez-Concha, T.^2,12^; Murueta-Goyena, A.^1,3^; Lafuente, J.V.^3^; Prada, Á.^4,10^; López de Munain, A. ^5,6,11,12,13^; Ruiz Irastorza, G.^7,14^; D. Martín-Iglesias^7,14^; Ribacoba, L.^8^; Gabilondo, I.^1,2,9^; Gómez-Esteban, J.C.^1,2,3,12,13^; Tijero-Merino, B.^1,2,12,13^.

1.Neurodegenerative Diseases Group, Biocruces Bizkaia Health Research Institute, Barakaldo, Spain.

2.Department of Neurology, Cruces University Hospital-OSAKIDETZA, Barakaldo, Spain.

3.Department of Neurosciences, University of the Basque Country UPV/EHU, Leioa, Spain.

4.Department of Immunology, Donostia University Hospital-OSAKIDETZA, San Sebastián, Spain.

5.Department of Neurology, Donostia University Hospital-OSAKIDETZA, San Sebastián, Spain.

6.Department of Neurosciences, Biodonostia Health Research Institute, San Sebastián, Spain.

7.Autoimmune Diseases Research Unit, Biocruces Bizkaia Health Research Institute, Barakaldo, Spain.

8.Department of Internal Medicine, Cruces University Hospital, Barakaldo, Spain.

9.The Basque Foundation for Science, IKERBASQUE, Bilbao, Spain.

10.Spanish Network for the Research in Multiple Sclerosis, San Sebastian, Spain.

11. Department of Neurosciences. University of the Basque Country UPV-EHU, San Sebastián, Spain.

12. Department of Medicine, School of Medicine, University of Deusto, Bilbao, Spain.

13. CIBERNED-CIBER, Institute Carlos III, Madrid, Spain.

14. Department of Autoimmune Diseases, Cruces University Hospital-OSAKIDETZA, Barakaldo, Spain.

***corresponding author:** Rocío Del Pino, Ph.D. Neurodegenerative Diseases Group, Biocruces Bizkaia Health Research Institute, Plaza de Cruces 12, Barakaldo (Bizkaia), CP 48903, Spain. Corresponding author’s phone: +34 946006000 ext. 7961. Corresponding author’s e-mail: delpinorocio@gmail.com

**corresponding author:** Juan Carlos Gómez Esteban, Ph.D. MD. Neurodegenerative Diseases Group, Biocruces Bizkaia Health Research Institute, Plaza de Cruces 12, Barakaldo (Bizkaia), CP 48903, Spain. Corresponding author’s phone: +34 946006000 ext. 7961. Corresponding author’s e-mail: juancarlosgomezesteban@gmail.com

**Table 1 Neuropsychologic and neuropsychiatric assessment**

| **Neuropsychologic assessment** | | |
| --- | --- | --- |
| **Composite** | **Cognitive domain** | **Test** |
| General cognition | General cognition | MoCA |
| Verbal fluency | Verbal fluency | Animals |
|  |  | P words |
| Processing speed | Visual processing speed | SDMT |
|  |  | SPCT |
|  | Visual processing speed,attention and inhibition | Stroop Test |
| Attention | Verbal attention | Direct digits (WAIS-IV) |
|  | Visual attention | TMT-A |
|  | Sustained attention | TP-R |
| Verbal memory | Verbal memory | HVLT-R |
| Visual memory | Visual memory | TCF memory |
|  |  | BVMT-R |
| Visuoconstructive ability | Visuoconstructive ability | TCF copy |
| Visuospatial perception | Visuospatial perception | Benton JLO |
| Abstraction | Abstraction | Similarities (WAIS-IV) |
| Executive functions | Working memory | Indirect digits (WAIS-IV) |
|  | Cognitive flexibility | M-WCSMT |
|  | Alternating attention | TMT-B |
| **Neuropsychiatric assessment** | | |
|  | Health perception | SF-36 |
|  | Sleep quality | PSQI |
|  | Fatigue | MFIS |
|  | Performance status | Karnofsky scale |
|  | Anxiety | Anxiety trait (STAI) |
|  |  | Anxiety state (STAI) |
|  | Depression | GDS |
|  | Suicidal ideation | C-SSRS |

Benton JLO: Benton Judgment Line Orientation; BVMT-R: Brief Visuospatial Memory Test-Revised; HVLT-R: Hopkins Verbal Learning Test-Revised; C-SSRS: Columbia Suicide Severity Rating Scale; GDS: Geriatric Depression Scale; GIAP: Global Index of Attention and Perception; ICI: Impulsivity Control Index; MFIS: Modified Fatigue Impact Scale; MoCA: Montreal Cognitive Assessment; M-WCST: Modified Wisconsin Card Sorting Test; PSQI: Pittsburgh Sleep Quality Index; SDMT: Symbol Digit Modality Test; SF-36: The 36-Item Short Form Health Survey; SPCT: Salthouse Perception Comparison Test; STAI: State-Trait Anxiety Inventory; TCF: Taylor Complex Figure; TMT: Trail Making Test; TP-R: Toulouse-Piéron-Revised Test; WAIS IV: Wechsler Adult Intelligence Scale IV.

|  | | **Autonomic Nervous System and hemodynamic parameters** | | | | | | | | | | | | | | | | | | |
| --- | --- | --- | --- | --- | --- | --- | --- | --- | --- | --- | --- | --- | --- | --- | --- | --- | --- | --- | --- | --- |
| **Post-COVID** | **HF-RRI** | **HFnu-RRI** | **LF-dBP** | **LFnu-dBP** | **LF/HF** | **SV** | **CO** | **TPR** | **BRS** | **Deep breathing index** | **E/I ratio** | **Valsalva ratio** | **Valsalva PRT** | **ΔsBP phase II late** | **ΔdBP phase II late** | **ΔsBP phase IV** | **ΔdBP phase IV** | **Supine HR** | **Mean HR tilt** |  |
| Sudoscan hands |  |  |  |  |  |  | .30** |  |  | .25* | .23* |  |  |  |  |  |  |  |  |  |
| Sudoscan feet |  |  |  |  |  |  | .32** |  |  | .22* |  |  |  |  |  |  |  | .25* |  |  |
| QST heat | .22* | .26* |  |  |  |  |  |  | .28** | .26* | .26* |  |  |  |  | .24* |  |  | .29** |  |
| QST heat pain |  | -.28** |  |  |  |  |  | .25* | -.31** |  |  |  |  |  |  |  |  |  |  |  |
| QST cold pain |  |  |  | .28** | -.23* |  |  |  |  |  |  |  |  |  |  |  |  |  |  |  |
| QST heat detection | .39*** | .30** |  |  | -.27* |  |  |  | .36*** |  |  |  |  |  |  |  |  |  | .22* |  |
| N latency (heat) |  |  |  |  |  |  |  | .26* |  |  |  |  |  |  |  |  |  |  |  |  |
| N amplitude (heat) |  |  | .25* |  |  |  |  |  |  |  |  |  |  |  |  | .24* | .30** |  |  |  |
| P latency (heat) |  |  |  |  |  |  |  | .25* |  |  |  |  |  |  |  |  |  |  |  |  |
| P amplitude (heat) |  |  |  |  |  |  |  | -.26* |  | .23* | .25* |  | -.26* |  |  |  |  |  |  |  |
| N latency (cold) |  | -.22* |  |  |  | -.23* |  |  |  |  |  |  |  |  |  |  |  |  |  |  |
| P latency (cold) |  |  |  |  |  | .25* | -.26* |  |  |  |  |  | .25* |  |  |  |  |  |  |  |
| **ME/CFS** |  |  |  |  |  |  |  |  |  |  |  |  |  |  |  |  |  |  |  |  |
| Sudoscan hands |  | -.32* |  | .44** | .36** |  |  |  |  | .37* | .41** | .32* |  |  |  | .39** |  | .31* | .35* |  |
| Sudoscan feet |  |  |  |  |  |  |  |  |  | .37** | .38** |  |  |  |  |  |  |  |  |  |
| QST cold |  |  |  |  |  |  |  |  |  | .29* | .41** |  |  |  |  |  |  |  |  |  |
| QST cold pain |  |  |  |  |  | -.35* |  | .35* |  |  |  |  |  |  |  |  |  |  |  |  |
| QST heat detection |  |  |  |  |  |  |  |  |  |  | .32* |  |  | -.51*** | -.36* |  |  |  |  |  |
| N amplitude (heat) | .37* |  |  |  |  |  |  |  | .29* |  |  |  |  |  |  |  |  |  |  |  |
| P amplitude (heat) |  |  |  |  |  |  |  |  |  |  |  |  |  |  | -.51*** |  |  |  |  |  |
| N amplitude (cold) |  |  |  |  |  |  |  |  |  |  |  |  |  |  | .36** |  |  |  |  |  |
| P latency (cold) |  |  |  |  |  |  | .31* | .29* |  | -.34* | -.33* |  |  |  |  |  |  | -.31* |  |  |

**Table 2 Autonomic Nervous System and hemodynamic parameter correlations with small fiber assessment**

*Note*: For Valsalva maneuver blood pressure data, the differences between the baseline and each phase were taken. **p* ≤.05; ***p* ≤.01; ****p* ≤.001. BRS: baroreflex sensitivity; CO: cardiac output; dBP: diastolic blood pressure; HF: high frequency; HR: heart rate; LF: low frequency; nu: normalized units; ME/CFS: Myalgic Encephalomyelitis/Chronic Fatigue Syndrome; PRT: pressure recovery time; RRI: R-R interval; sBP: systolic blood pressure; SV: stroke volume; TPR: total peripheral resistance.

**Table 3 Autonomic Nervous System and hemodynamic parameter correlations with neuropsychological assessment**

|  | **Autonomic Nervous System and hemodynamic parameters** | | | | | | | | | | | | | | | |
| --- | --- | --- | --- | --- | --- | --- | --- | --- | --- | --- | --- | --- | --- | --- | --- | --- |
| **Post-COVID** | **HF-RRI** | **HFnu-RRI** | **LF-dBP** | **LF/HF** | **SV** | **BRS** | **Deep breathing index** | **E/I ratio** | **Valsalva ratio** | **Valsalva PRT** | **ΔdBP phase II late** | **ΔsBP phase IV** | **ΔdBP phase IV** | **Supine HR** | **Mean HR tilt** |  |
| General cognition | .27* | .26* |  |  |  | .26* |  | .26* |  | .25* |  |  |  |  |  |  |
| Verbal fluency | .31** | .28** |  |  |  | .26* |  |  |  |  |  |  |  |  |  |  |
| Processing speed | .31** |  | .24* |  | .25* |  |  |  |  |  |  |  |  |  | -.36*** |  |
| Attention | .25* |  |  |  |  |  |  |  |  |  |  |  |  |  | -.32** |  |
| Verbal memory | .25* | .28** |  | -.25* |  |  | .27* | .25* |  |  |  |  |  |  |  |  |
| Visual memory | .32** | .31** |  | -.24* |  | .33** | .28* | .29** |  |  |  |  |  |  |  |  |
| Visuospatial perception | .23* |  |  |  |  |  |  |  |  |  |  |  |  |  |  |  |
| Abstraction |  |  |  |  |  |  |  |  |  | .24* |  |  |  |  |  |  |
| Executive functions | .22* | .25* |  | -.24* |  |  |  |  |  |  |  |  |  |  |  |  |
| SF-36 | -23* |  |  |  | -23* |  |  |  |  |  | .33** |  |  |  | -.23* |  |
| PSQI | .23* |  |  |  | .23* |  |  |  |  |  |  |  |  |  |  |  |
| MFIS |  |  |  |  | -.25* |  |  |  |  |  |  |  |  |  |  |  |
| Karnofsky scale |  |  |  |  | .22* |  |  |  |  |  | .28* |  |  |  |  |  |
| GDS |  |  |  |  |  |  |  |  |  |  |  |  |  |  | .24* |  |
| **ME/CFS** |  |  |  |  |  |  |  |  |  |  |  |  |  |  |  |  |
| General cognition |  |  |  |  |  |  |  |  |  |  |  | .39** |  |  | .35* |  |
| Verbal fluency |  |  |  |  |  |  |  |  |  |  |  |  |  |  | .31* |  |
| Processing speed |  |  |  |  |  |  |  |  | .39** |  |  | .32* |  |  |  |  |
| Attention |  |  |  |  |  |  |  |  | .34* |  |  |  |  |  |  |  |
| Verbal memory |  |  |  |  |  |  |  |  |  |  |  |  |  | .29* | .37** |  |
| Visual memory |  |  |  |  |  |  | .30* |  | .37** |  |  |  |  |  |  |  |
| Visuoconstructive ability |  |  |  |  |  |  |  |  | .37** |  |  | .38** |  |  |  |  |
| Visuospatial perception |  |  |  |  |  |  |  |  |  |  |  | .31* |  |  | .29* |  |
| Executive functions |  |  |  |  |  |  |  |  | .34* |  |  |  |  |  |  |  |
| SF-36 |  |  |  |  |  |  |  |  | .31* |  |  |  |  |  |  |  |
| PSQI |  |  |  |  |  |  |  |  |  |  | -.47** | -.31* |  |  |  |  |
| GDS |  |  |  |  |  |  |  |  |  |  |  |  | -.36* |  |  |  |

*Note*: For Valsalva maneuver blood pressure data, the differences between the baseline and each phase were taken. **p* ≤.05; ***p* ≤.01; ****p* ≤.001. BRS: baroreflex sensitivity; CO: cardiac output; dBP: diastolic blood pressure; HF: high frequency; HR: heart rate; LF: low frequency; nu: normalized units; ME/CFS: Myalgic Encephalomyelitis/Chronic Fatigue Syndrome; MFIS: Modified Fatigue Impact Scale; PRT: pressure recovery time; RRI: R-R interval; PSQI: Pittsburgh Sleep Quality Index; sBP: systolic blood pressure; SF-36: The 36-Item Short Form Health Survey; SV: stroke volume; TPR: total peripheral resistance.
